# A faculty development workshop in narrative-based reflective writing

**DOI:** 10.1007/s40037-012-0021-4

**Published:** 2012-08-08

**Authors:** J. Donald Boudreau, Stephen Liben, Abraham Fuks

**Affiliations:** 1 Department of Medicine, McGill University, 3655 Drummond St., McIntyre Medical Sciences Building, Room 529, Montreal, QC H3G 1Y6 Canada; 2 Department of Pediatrics, McGill University, 2300 Tupper St., Montreal Children’s Hospital, Montreal, QC H3H 1P3 Canada; 3 Department of Pediatrics, McGill University, Montreal, QC Canada; 4 Department of Medicine, McGill University, 3647 Peel St, Montreal, QC H3A 1X1 Canada

**Keywords:** Narrative, Creative writing, Reflective writing, Self-reflection, Faculty development

## Abstract

Narrative approaches are used increasingly in the health professions with a range of objectives. We must acquaint educators with this burgeoning field and prepare them for the incorporation of story-telling in their pedagogical practices. The authors describe a template for a faculty development workshop designed to foster self-reflection through the use of narrative techniques and prepare clinical teachers to deploy such approaches. The design is based on a six-year experience in delivering introductory workshops in narrative approaches to medical teachers. The workshops, which served as a model for the template, have been offered to a total of 92 clinicians being trained to mentor medical students. A generic template is described. It includes a table of core concepts from narrative theory, a set of probing questions useful in a basic technical analysis of texts and a list of initiating prompts for exercises in reflective writing. A workshop organized and deployed using this template is deliverable over a half-day. The model has proven to be feasible and highly valued by participants. It can be adapted for other contexts by educators across the continuum of health professional education.

## Introduction

Over the past three decades, narrative theory has become increasingly prominent in the health professions. This trend has coincided with a similar phenomenon in the humanities where it has been referred to as the ‘narrativist turn’ [1]. Narrative in the context of medicine has acquired diverse forms and functions. One of the earliest versions of continuing professional development grounded in story telling was the Balint groups [2]. First implemented in 1950, they were designed to support the doctor–patient relationship by focusing attention on physicians’ emotions arising out of clinical encounters, particularly those perceived as puzzling or unsettling. Although the Balint group method did not specifically revolve around an understanding of narrative as a genre, stories—in the guise of ‘cases’—were the indispensable ingredient [3]. A similar activity has been termed ‘reflective writing’ in that written stories serve as stimuli to and the subject matter for individual or group reflection. In the UK, reflective writing courses have been deployed for the on-going professional education of general practitioners [4]. Participants are urged to write about events or ideas in their personal and professional lives that are troubling or hard to resolve and to share these stories with peers in a supportive group setting.

In essence, ‘narrative’ is ‘a story and its telling’. We use the terms narrative and story interchangeably. Narrative is manifest in many forms in addition to the written. Stories constitute the basis of psychotherapeutic intervention and narrative has evolved as a stream within bioethics. Many health care professionals will recognize it as a specific methodology in qualitative research. In order to capture the breadth of expression of narrative within medicine, a taxonomy was recently constructed and published [5]. A notable entry in this taxonomy is ‘narrative medicine’. The term was coined and the subject given much of its character and appeal by the physician and literary scholar Rita Charon. She views it as a clinical method that makes explicit use of narrative skills and defines it as: ‘[a] *medicine practiced with the narrative competence to recognize, interpret, and be moved to action by the predicaments of others*’ [6]. That is a rather expansive conception of narrative within medicine. There are alternative notions in which narrative is considered less a defined clinical discipline and more a theoretical concept with instrumental possibilities. There is an expanding literature on the application of narrative strategies. For example, narrative-based programmes designed to promote ‘ethical mindfulness’ have been outlined in some detail [7, 8]. Despite these innovations, we are not aware of a description of an introductory faculty development workshop in narrative-based reflective writing that would be appropriate for clinical teachers.

The foundational premise for the use of contemplative writing in medical education is that the very act of putting one’s storied experiences down on paper, of selecting the precise manner in which to recount a tale (e.g. choosing which characters to include and which to leave out; deciding which of several constitutive events should serve as the beginning, middle and end; adopting a particular point of view; creating an emotional tone) activate introspection and critical reflection. An added benefit is that the written text, in comparison with a spoken monologue or colloquy, is enduring; it is a ‘thing’ that, having assumed a certain form is then available for subsequent review and analysis. It has been claimed that reflective writing can meet a variety of objectives: the list includes the nurturing of empathy [9], training for mindfulness [10], professional identity formation [11], and prevention of burnout [12]. Whether reflective writing actually accomplishes such laudable goals, and how it does so, are essentially unknown. It has been hypothesized that there is a ‘transfer effect’ whereby skills in story-writing, listening and recounting are generalizable to alliance building with patients [13]. One teacher who uses illness narratives to foster empathy suggests that they do their work by creating a state of emotional or cognitive disequilibrium [14]. The conceptual foundations of narratology and the theories on how writing comes to be in the service of reflection are beyond the scope of this essay.

Our aim is to describe the core elements of a faculty development workshop in narrative-based reflective writing. While we were guided by the needs of teachers in an undergraduate medical programme, our intent is not to propose a template that is narrowly focused on one particular audience. We believe there is a need for a template that is feasible, scalable, adaptable to the continuum of health professional education and respectful of the multiplicity of concepts attributable to narrative in medicine. We begin by describing a locally based programme and then expand the scope to a potentially larger audience by broaching issues integral to the design of any faculty development programmes of this nature.

## Method

The template we designed is based in a six-year experience (2005–2011) of conducting introductory workshops in narrative at McGill University’s Faculty of Medicine. More specifically, it arises from a retrospective critical review process of ‘meta-evaluation’ which, according to the 3rd edition of ‘Programme Evaluation Standards’ of the Joint Committee on Standards for Educational Evaluation, is a ‘self-awareness, pausing, openness to monitoring, scrutiny, and a changing of course’ [15]. This definition captures our longitudinal experience with these workshops and our resultant reflections. We benefited from the involvement of experts in the field, including Rita Charon who served as facilitator at our inaugural session. We modified the workshops iteratively based on participant feedback and developed new audiovisual materials as aids to learning.

The sessions were conducted over a half day and delivered to clinical teachers in a formal mentorship programme for medical students. This programme, entitled ‘Physician Apprenticeship,’ has two primary goals: (i) to assist students in their transition from laymanship to physicianship to become professionals and healers, patient-centred and reflective practitioners, and (ii) to provide a safe and supportive environment where students are encouraged to discuss issues arising out of professional socialization and their educational experience. The apprenticeship groups consist of six students, one or two senior medical students and a clinical teacher. These individuals (with the exception of the student co-leaders, who graduate approximately half-way through the programme) remain constituted as a group for the duration of the medical school curriculum of 4 years. They meet approximately five times per year. The clinical teachers, who are called Osler Fellows, are offered a targeted faculty development programme that aims to equip them to meet the objectives of the apprenticeship [16]. Three to four workshops are held annually; the series includes sessions on small group facilitation skills, mindfulness, cultural sensitivity training and narrative in medicine. The session on narrative serves three purposes: (i) to introduce basic narrative theory and precepts, (ii) to practice reflective writing using narrative as a frame for a shared dialogue and analysis, and (iii) to discuss strategies that Osler Fellows might consider using in integrating the reflective writing exercises within their apprenticeship groups. All three objectives are necessarily intertwined. Up to 2011, we welcomed 92 participants (approximately 16 per session) to our narrative medicine workshops. The template we describe stems from a long-standing tradition of effective small group facilitation, and was informed by the experience of two of the authors (AF, SL) in using narrative techniques in medical humanities electives.

## Results

### A practical template for an introductory workshop in narrative-based reflective writing

Our session is organized in four sections: introduction, a didactic portion, practical exercises and debriefing. In the introduction, we review the three session objectives, as outlined above, and the workshop agenda. In the didactic portion we define narrative, explain how it differs from non-narrative texts and present concepts in narrative theory with an emphasis on basic textual analysis. We do this quite rapidly, not wishing to compress the time allocated to the practical exercises. We explain that narrative can be considered an epistemology—a way of knowing the world that is complementary to the logico-scientific mode [17]. We found this contextualization to be very attractive to clinicians who, although they tacitly operate within both modes, may not have considered their quotidian work in this framework. We also define the following: causality, timeline, voice, point of view, genre, character and plot. Table 1 provides a list of these basic concepts, culled from standard works, notably ‘The Cambridge Introduction to Narrative’ [18].

**Table 1:** Basic concepts of narrative structure with examples of questions useful in exploring these concepts

Concept	Illustrative probing questions
Genre	*What literary type (or genre) do you think this writing might be described as (e.g. prose, poetry, drama, obituary, prayer, diary entry, legal document, recipe,* etc*.)?*
Narrator vs. author	*Do we know who the author is? Who is the narrator?*
Narration	*Is the voice of the narrator in the 1st person (I), 2nd person (you), 3rd person (he/she/them)? What is the narrator’s status (e.g. powerful, expert, novice, victim, neutral, biased, involved, distant)? Does the narrator change during the course of the text? Are there multiple points of view (POV) expressed in the narration? Example: Is there a third*-*person omniscient* *POV? Is such a POV possible? How would you describe the visual lenses (or focalization) through which you are apprehending the events and characters in this narration?*
Diction	*Is it serious, grandiose, cold/impersonal, pleading, clinical, casual,* etc*.?*
Time	*When does this story occur? Is it in the past, present or future or a combination? How much time passes in the story? In what order are events described? Are there any flashbacks or flash*-*forwards?*
Plot	*What happens in this story? Can you recognize a type of story that this narrative may resemble? Have you been exposed to this theme before (e.g. story of quest, chaos, revenge, love, restitution?)*
Images	*What images are conjured up? What metaphors are used? What do you wonder about?*
Feelings/emotions	*What feelings are evoked in this text?*
Gaps or ‘left out text’	*What might be ‘missing’ from the text? Is there anything you want to know more about in this story?*
Meaning	*What meanings do you think the author is trying to convey in this story?*

*Note* this table is based on *The Cambridge Introduction to Narrative* by H. Porter Abbott [18]

The session unfolds with a presentation of ground rules—a set of ‘must do’s’ and ‘must never do’s. We insist on the following three points: (a) confidentiality must be respected; (b) sharing of stories with the group is voluntary; and, (c) mutual respect is critical. We state: *What we write and discuss are not to be shared with others outside the group. It is strictly confidential. Although participation in writing exercises is required it is not obligatory to disclose what has been written. No one has to share anything should he or she feel uncomfortable in so doing. The option to ‘pass’ on sharing narratives with the group is always available. Respect the person who is speaking even if you disagree with what is said.* We emphasize these ground rules and urge the Osler Fellows to pay particular attention to them in narrative-based writing exercises with their students in the Physician Apprenticeship course.

Next, in preparation for the practica, the cohort of Osler Fellows is subdivided into smaller groups (comprising 4–6 individuals). We emphasize that the purpose of the practica is to examine how closely we (collectively) listen, connect and respond to each other rather than being about how well we can read and/or write. This statement helps to allay apprehensions since many health professionals are self-conscious about their literary prowess. We assign roles and give role-specific instructions to participants. In this generic template there is a story-teller (narrator) and story-receivers.

We have found the creation of triggers or discussion prompts to be the most demanding aspects of session design. The prompts need to be unambiguous and brief, to the point of being pithy. As an introduction to reflective writing we rely on instructions often used by Rita Charon in the workshops she facilitates. Two such examples are: (i) ‘*Write about your name*—*anything you want to say about your first or family name’* and, (ii) ‘*Write about one of your scars.’* It is also very important that the writing prompt be appropriate to the participants’ professional and personal experiences. Asking medical students who are in a pre-clinical phase of their programme to write about their emotional reactions to patients when they have had little clinical exposure would be inappropriate and frustrating and may instil a future bias against similar exercises. For students at this level it may be more productive to focus on issues that routinely preoccupy them such as test or exam anxiety and emotions arising out of negative assessments. As students are initiated in the clinical phase, a focus on their emergent professional identity may be fruitful; for example, an exploration of their first experience with a dying patient or their first clinical mistake would almost certainly provoke introspection. A list of potentially useful writing prompts is provided in Table 2. With each of these triggers we caution the participants not to deliberate too much about what to compose but rather to be spontaneous and to write about the first things that come to mind. We generally allocate 5–10 min for the writing. Another option is to provide participants with part of a short story (generally less than a page), with the ending omitted, and then ask them to write a conclusion to the story. Regardless of the writing task we avoid predetermining the direction of the subsequent group discussion. The following instruction will illustrate that point. It is preferable to say, *‘Write about the first time one of your patients died’* rather than *‘Write about the first time one of your patients died so that we can explore how this event contributed to your maturation as a clinician.’* The latter formulation is too constraining and may dampen creativity and hinder imaginative leaps.

We then invite a volunteer to read aloud to the group the text he or she has written, exactly as it was written down. In order to respect the written text, facilitators gently intercept or redirect editorializing back to the reading of what was written, as there is a natural tendency to want to explain or justify one’s choice of words. The first question is always directed at the author. Examples of opening questions are: *‘Is there anything you’d like to share with us about why you chose this event?; ‘What is this story trying to tell?’* We then engage in a small group discussion of the text as viewed through a narrative lens while avoiding profound psychoanalytic interpretations of what has been written. For example, the facilitator might ask the group: *‘The story was written in the 3rd person; does that influence how you connect or do not connect with the main character?’* Alternatively, the facilitator may challenge the reader with: *‘The rhythm of your text is very staccato, written as if it were in point form; can you tell us why you used this format?’* or, *‘The tone seems rather cerebral or intellectual to me; do others agree? Did you feel this way when you were in the actual encounter*?’ Gaps or silences in stories can be particularly revealing; they often mark unresolved tensions. In such a circumstance a gentle prompt such as, *‘There was a long pause there before you jumped backwards in time; do you know what was going on in your mind during that pause?’* A menu of such probes is provided in Table 1. Examples are also available in descriptions of other narrative-based workshops such as the one previously referenced on teaching ethical mindfulness [8]. The aim is to guide the writer and the group in a structured reflection on something meaningful that they heard. The ultimate purpose is to generate a multifaceted, layered and triangulated understanding that emerges from the interplay of the written text and the group discussion. Our session facilitator(s) conclude the practica by inviting final comments from the writer who has just shared a narrative with the group. Any raw emotions remaining are dealt with or, at a minimum, acknowledged for follow-up at a later time, as appropriate. The smaller groups then reconvene as a larger group.

**Table 2:** A list of writing prompts

‘Ice-breaker’ type of prompts—to be used at the beginning of the workshop:	*Write about your name*
	*Write about one of your scars*
	*Describe something important in your life: a pet, a hobby, a car, a house, a song—anything except a person*
Writing prompts that may be appropriate for clinical teachers or practitioners:	*Think of a clinical encounter that involved suffering and write a description of it. You may write about any aspect: what you thought and felt or what you think the patient or family thought and felt*
	*Write about a clinical encounter in which you regret your action*
	*Write about an episode where you made an error, or one in which you triumphed*
Writing prompts that may be appropriate for students in the health professions:	*Describe your first…* [first experience of a patient dying] [first time dissecting a cadaver] [first time you got angry with a patient] [first time you felt incompetent] [first assist at a cardiac arrest] [first time you cried in a clinical setting] [first time you felt like a doctor] [first time you felt like throwing in the towel] [first time on call] [first time you physically hurt a patient (e.g. invasive procedure)]
	*Write about a patient encounter episode where you witnessed healing or where there was a missed opportunity to provide healing*
	*Describe an event where you suddenly realized that you are becoming a* [doctor] [nurse] [midwife] [dentist]

The workshop closes with an attempt at synthesis and an invitation to the Osler Fellows to consider how they might apply the technique of reflective writing, using narrative as a guiding framework, in their student groups. Finally, we solicit feedback and recommendations for improvement. As with any tutorial, the session is evaluated using standard anonymous questionnaires which incorporate both quantitative and qualitative data. It is preferable that the evaluation be conducted immediately upon closure rather than at a later date.

### Evaluation of the prototype workshop

A framework often used in programme evaluation is Kirkpatrick’s four-level model: reaction, learning, performance, and results [19]. Descriptions of outcomes of programmes in narrative have generally limited themselves to the first level, i.e., participant reaction. The strategy we deployed for evaluating our annual workshops is of that nature. We confined ourselves to Kirkpatrick level 1 and our quantitative data focused on participant satisfaction. We used a questionnaire based on a Likert scale with five response categories, where 1 = not at all useful; 3 = useful; and 5 = very useful. Of the 92 participants, 83 completed the questionnaire (response rate 90 %). The respondents were slightly more satisfied with the practical exercises than the didactic portion of the workshop. With respect to the didactic segment, 50 % of the respondents selected ‘4’ and 40 % selected ‘5’. With respect to the practical exercises, 34 % selected ‘4’ and 63 % selected ‘5’. The qualitative section was organized around three themes; we wanted to understand which aspects of the workshop were most and least useful and solicited recommendations for change. The written comments were invariably supportive. The following were typical: *‘I was a bit apprehensive about this topic to start off with. I personally do not like to write down my thoughts but, in the end, I was surprised to see that I actually enjoyed doing so.’ ‘Understanding narrative medicine, realizing that we think and listen that way, is very useful.’* The most prevalent specific recommendations revolved around ensuring that in future workshops everyone should be accorded the opportunity to share stories: ‘*Extend the length of the practicum so everyone has time to read his narrative.’ ‘If you’re going to ask us to write something, there should be a built*-*in opportunity to share what was written. It is anti*-*climactic otherwise.’* A second cluster of recommendations had to do with the quality of the writing triggers: ‘*Experiment with positive emotions; don’t write about suffering—choose an uplifting topic.*’ *‘The name*-*writing exercise was a fun way to illustrate the concept, but redundant given the exercises afterwards.’*

We report these findings since we feel that our recommendation for a generic template would otherwise be weakened. We have used participant feedback to refine our design in an iterative manner and, most importantly, to enrich our bank of triggers or prompts for writing exercises and the probes used in textual analysis. Informal feedback by the Osler Fellows, given to us in the context of other (non-narrative) faculty development workshops, suggests that while many of them have tried it in their student groups, it has not always been easy or totally successful. Several commented that their trials were stressful because the sessions evolved into a kind of ‘group therapy’ and they felt neither experienced nor interested in engaging in informal psychotherapy. Interestingly, even those who found the experience difficult were not ready to abandon it; many have requested review sessions or lengthier workshops (e.g. full day instead of half-day). Ideally, our approach to workshop evaluation should mirror the fact that the session had three separate objectives: getting acquainted with narrative theory, learning skills in self-reflection by means of narrative, and learning how to use these methods and skills as mentors. We plan to modify the questionnaire accordingly in future sessions.

### Theoretical considerations

Our local experience with narrative in the service of teaching and role-modelling self-reflection within the context of a mentorship programme in undergraduate medicine inspired the template we have just described. As underlined in the introduction, we hope that it will be seen as relevant to a broader audience. We do not wish the template to be overly prescriptive. We therefore invite readers to modify it to their own needs. In doing so, the following issues should be considered.

The initial steps in planning any faculty development workshop are to identify the audience and the primary objectives. As previously noted, narrative techniques have been applied to a variety of educational goals in diverse contexts. Session organizers need to consider if there are other curricular models or specific methodologies that might constitute a useful guide. The experiences of other programmes can be invaluable. A programme of interactive reflective writing for undergraduate medical students at Brown University makes use of the cognitive apprenticeship model; the tools it employs, with structured field note prompts, may be applied to other contexts [20]. Another example is the programme in clinical supervision of family physicians offered at the Tavistock clinic in London; it blends narrative and systems theory while bridging theory and application through the use of specific interview techniques [21]. The narrative-based clinical supervision sessions conducted at the Tavistock clinic often proceed with five generic roles: a narrative dyad (i.e. a client physician as story teller and an interviewee as interlocutor); an observer of the narrative dyad; a facilitator of the supervisory session and a reflective team (of variable numbers). This protocol may prove useful for a more advanced workshop whose aim is to deepen participants’ understanding of their roles and personas in the doctor–patient relationship. A protocol which aims to mitigate health provider burnout combines a narrative approach with appreciative inquiries and self-awareness exercises [22]. In contrast to these highly sophisticated exemplars, an introductory workshop should have modest objectives. It should be practical and unfold with an easily administered protocol. We trust that our proposed template fulfils that need.

Specific attention must be accorded to the type of narratives that will be utilized. While there is a tendency to conflate narrative with written stories, one must keep in mind that there are non-written narrative forms. Stories may be verbally communicated or remain confined to an internal monologue. They can be acted as well as written or told—in movies, plays, electronic storyboards or comic books. They can be produced prior to a workshop or written in real-time during a session. Stories can also be accessed from the literature; many readers will be aware of the honoured tradition of using classic literature in medical education [23]. There are two distinct approaches for working with written texts in the health professions: close reading and creative writing. The close reading of narratives previously written by patients or insightful clinicians is useful for a workshop whose aim is to reveal patients’ perspectives on the experience of illness. Creative writing is particularly suited to stimulate reflection. As we have seen, the raison d’être of our workshops was to promote self-reflection; we therefore depended on written materials generated by participants themselves in the course of the actual meetings. These texts then served as materials for discussion and analysis by fellow participants.

Many aspects of group dynamics are germane to narrative work and facilitators must be versed in the generic principles of small group teaching. They must assiduously avoid interruptions and limit their own speaking time; encourage, reassure, question reflectively; attend to participants’ emotions; diffuse any confrontations; and create opportunities to be positive and supportive. They must actively promote interaction. It is critical that they withhold detailed interpretations, commentaries, judgments and editorial pronouncements. They must attend to important ‘house-keeping’ items such as timing and writing exercises. Of utmost importance is an ability to listen attentively; while it is often underscored, there are few descriptions of what this entails in a practical sense. The actions of workshop facilitators (for example, what is said, how it is said, what is not said) are vehicles for modelling listening skills for participants. Simply talking about the importance of listening will have little impact. Active listening involves paying attention non-judgmentally to both the speaker’s and one’s own internal dialogue, to the creation of comfortable spaces for silence, to openness to others and to the skilful delivery of clarifying questions. Active listening is best taught by example. It is critically important to an interactive faculty development session of any type.

## Conclusion

There is a growing appreciation of the importance in the health professions of reflective practice. As the educational focus has shifted towards understanding and supporting professionalization and identity formation it has been paralleled by an expanding interest in strategies, such as narrative skills, which can provide an entry into the process. These developments are anchored in the idea, expressed so eloquently by neo-Aristotelian philosopher Alasdair MacIntyre that ‘the chief means of moral education is the telling of stories.’[24].

Health care professionals are very receptive to the belief that an acquisition of narrative skills can contribute to more effective and critical reflection and enhance communication with peers, patients and learners. Despite the centrality of stories to many of the tasks that clinicians perform it remains that explicit and formal teaching of knowledge and methods in narrative is relatively novel. The template we have presented for an introductory faculty development workshop is not intended to be comprehensive nor should it be received as a procrustean operational rule book. Rather, it is meant to provide a basic overview, anchored to reflective writing as one particular expression and use of narratives. It is clear to us that if this emerging discipline is to fulfil its various promises additional tools for faculty development as well as longitudinal programmes in evaluation will be required. The template we propose, based on a successful practice with clinical teachers engaged in a mentorship programme, is a modest contribution to that end.

## Essentials

- Narrative theory and narrative methods have been increasingly integrated throughout the continuum of health professional education and used specifically to nurture self-reflection.
- Skills in narrative-based reflective writing can be introduced successfully to clinical teachers through a brief faculty development workshop.
- An interactive workshop, deliverable over a half-day, has been well received by participants although long-term outcomes on effectiveness and impact are lacking.
- A generic template for designing and conducting an introductory workshop has been described.
- The most challenging aspect in the design of such a narrative-based workshop is the crafting of appropriate triggers for the writing exercises.
